# Impact of crestal and subcrestal implant placement upon changes in marginal peri-implant bone level. A systematic review

**DOI:** 10.4317/medoral.23006

**Published:** 2019-08-19

**Authors:** Hilario Pellicer-Chover, María Díaz-Sanchez, David Soto-Peñaloza, María Peñarrocha-Diago, Luigi Canullo, David Peñarrocha-Oltra

**Affiliations:** 1 DDS, PhD. Collaborating Professor of the Master in Oral Surgery and Implant Dentistry, Oral Surgery Unit, Department of Stomatology, Faculty of Medicine and Dentistry, University of Valencia, Spain; 2 DDS, Master in Oral Surgery and Implant Dentistry, Department of Stomatology, Faculty of Medicine and Dentistry, University of Valencia, Spain; 3 MD, PhD, DDS. Full Professor, Oral Surgery Unit, Department of Stomatology, Faculty of Medicine and Dentistry, University of Valencia, Spain; 4 DDS, PhD. Visiting Professor in Oral Surgery and Implantology, Stomatology Department, Faculty of Medicine and Dentistry, University of Valencia, Valencia, Spain; 5 DDS, PhD. Assistant Professor, Oral Surgery Unit, Department of Stomatology, Faculty of Medicine and Dentistry, University of Valencia, Spain

## Abstract

**Background:**

To systematically assess studies analyzing peri-implant bone loss in implants placed in crestal and subcrestal position.

**Material and Methods:**

Following the recommended methods for systematic reviews and meta-analyses (PRISMA), an electronic search was conducted in the PubMed (MEDLINE), EMBASE and LILACS databases to identify all relevant articles published up until April 2017. The search included human studies comparing marginal bone loss (MBL) between a control group and a study group with a minimum of 10 patients and a minimum follow-up of 6 months after prosthetic loading with rough neck implants. Two independent reviewers assessed the risk of bias in the selected studies based on the Newcastle-Ottawa scale for observational studies and the Cochrane Collaboration for clinical trials.

**Results:**

Of 342 potentially eligible items, 7 complied with the inclusion criteria. One article was retrieved through the manual search. Eight articles were finally included: five experimental and three observational studies. The risk of bias assessed by the Cochrane Collaboration and Newcastle-Ottawa showed a high risk of bias. The mean follow-up period was 21 months (range 6-36 months). In four studies, implants placed in a crestal position presented higher MBL than subcrestal implants - the differences being significant in one study, while in three studies, implants placed in a subcrestal position presented greater MBL than crestal implants, with significant differences in only one study.

**Conclusions:**

Despite its limitations, the present systematic review did not find better outcomes between crestal and subcrestal implant placement, however, new studies will be needed, involving improved designs and the standardization of protocols to allow statistical comparisons and the drawing of firm conclusions.

** Key words:**Crestal implants, subcrestal implants, placement level, systematic review.

## Introduction

Many authors (1-3) have observed peri-implant bone losses of between 1-2 mm after the first year of occlusal loading, and of 0.1 to 0.2 mm over successive years. Such bone loss has been associated to many factors, such as the periodontal biotype (4), the distance between implants (5), macro- and micro-implant design (6), and occlusal overloading (7). An additional factor is the presence of a microgap prone to microbial contamination in the implant abutment connection and, consequently, the location of this connection in relation to the bone crest (8-10).

Placement of an implant in a deeper position with respect to the bone crest (subcrestal placement) has been suggested as a method that could contribute to maintain the periimplant soft and hard tissues in comparison with crestal placement, though this affirmation is subject to controversy. As early as 1969, Branemark (11) recommended placing the implant below the bone crest to prevent implant exposure during bone remodeling. Some authors (10,12) have reported that implants placed approximately 2 mm below the bone crest are associated with significantly less peri-implant bone loss compared to implants placed at crestal level. Conversely, other authors (13-18) have observed greater bone loss with implants placed at subcrestal level. Variations in study design, implant geometry, surface treatment and surgical protocols in implant placement could explain the discrepancies in the results of the aforementioned studies.

Initial bone loss from implants has been associated with peri-implant bone loss over the long term and therefore to periimplantitis (19). At present, there is no consensus on optimal interventions for the treatment of peri-implantitis (20). Therefore, all implant maintenance programs are focused on prevention, that is, meticulous oral hygiene practices, careful peri-implant examination, analysis of risk factors and periodic elimination of bacterial deposits from implants (21). In this line, limiting the exposure of the rough surface of the implant could be relevant to maintain a correct long-term peri-implant health. Evidence suggests that the apicocoronal position influences bone loss, though there are conflicting opinions in this respect. Hence the aim of this investigation was to systematically review studies analyzing peri-implant bone loss in implants placed in crestal and subcrestal position.

## Material and Methods

The present systematic review was conducted in accordance with the PRISMA (Preferred Reporting Items for Systematic Reviews and Meta-Analyses) guidelines.

-Focus question

The present systematic review is therefore justified, with the aim of answering the following pre-specified focus question developed in accordance with the recognized Population, Intervention, Comparison, Outcome (PICO) format: “Are there differences in terms of marginal bone loss (MBL) between the subcrestal and juxta-crestal placement of osseointegrated implants in patients subjected to dental implant treatment?”

-Search strategy

An electronic search was performed without language and time restrictions and up until April 2017 in three main databases: the MEDLINE from the United States National Library of Medicine (NLM) through PubMed, EMBASE and LILACS. The medical subject “MESH” terms for PubMed, “EMTREE” for Embase and other free-text terms were used and combined whenever possible in each database.

In addition, electronic screening of the “grey literature” through the System for Information on Grey Literature in Europe (SIGLE) - Open Grey (http://www.opengrey.eu/) was performed as suggested by the AMSTAR guideline (22), attempting to minimize potential publication bias.

The search strategy in the PubMed database was conducted as follows.

((((“Dental Implants”[Mesh] OR “Dental Implants, Single-Tooth”[Mesh] OR dental implants OR titanium implants OR osseointegrated implants)) AND (subcrestal implants OR submerged implants)) AND (crestal implants OR non-submerged implants OR non submerged implants OR equicrestal implants OR juxta-crestal implants)) AND (“Bone Resorption”[Mesh] OR crestal bone loss OR marginal bone loss).

-Inclusion and exclusion criteria

 The following criteria were established to select articles for inclusion in the present review.

- Randomized clinical trials (RCTs), controlled clinical trials and prospective/retrospective cohort studies comparing crestal and sub-crestal implant placement

- Assessment of MBL

- Inclusion of at least 10 patients

- A minimum follow-up of 6 months after prosthetic loading

- Only studies including implants with a rough neck, and with or without platform-switching designs

Literature or narrative reviews, case-control studies, cross-sectional studies, case series, case reports, preclinical and in vitro studies, letters to the editor were excluded

-Data collection, extraction and management

-Screening and selection of papers

Titles and abstracts of potentially selected records were independently screened by two reviewers (H.P.C and M.D.S). Full reports were obtained and reviewed independently for studies that seemed to meet the inclusion criteria. To calibrate the interviewer reliability, percentages of agreement and Cohen’s Kappa coefficients were calculated. Disagreements between the authors were resolved following discussion and, if unresolved, another researcher (D.P.O.) could be consulted to reach consensus.

-Selection of studies and data extraction

The studies that met the inclusion criteria were processed for data extraction, which was conducted by two independent researchers (H.P.C. and M.D.S.). The following data were extracted and recorded in duplicate: author(s), year of publication, study design and details of the participants, intervention(s), MBL and relevant outcomes. Predefined data collection spreadsheets were employed for the assessment of each publication, and disagreements were resolved by discussion with a third reviewer (D.S.P.). In the event of missing data, a request was sent to the authors, if any.

-Risk of bias in individual studies 

Two independent reviewers (H.P.C. and M.D.S.) evaluated all the included articles. The methodological quality of observational studies was assessed with the Newcastle-Ottawa Scale (NOS) (23), and the Cochrane Collaboration tool for assessing the risk of bias was employed for the assessment of randomised controlled trials (24).

For each aspect of quality assessment, we scored the risk of bias following the recommendations of the Cochrane Handbook for Systematic Reviews of Interventions 5.1.0 (http://handbook.cochrane.org). Each entry was judged as “yes” (low risk of bias), “no” (high risk of bias) or “unclear” (either lack of information or uncertainty over the potential for bias).

The criteria included assessment of the followed items: 1) randomization and allocation methods (i.e., selection bias); 2) completeness of follow-up period/incomplete outcome data (i.e., attrition bias); 3) masking of patients (i.e., performance bias); 4) masking of examiners (i.e., detection bias); and 5) selective reporting (i.e., reporting bias). Based on these answers, risk of bias was categorized as: 1) low risk of bias if all criteria were met (i.e., adequate methods of randomization and allocation concealment, a ‘‘yes’’ answer to all questions about completeness of follow-up and masking of examiners, and a ‘‘no’’ answer to selective reporting and other sources of bias); 2) unclear risk of bias if one or more criteria were partially met (i.e., unclear criteria were set); or 3) high risk of bias if one or more criteria were not met. In addition, we developed a summary of bias appraisal to explain the reasons underlying judgment for each domain across studies (supplementary data file).

In cohort studies, each item of the scale could be awarded one point rated on a scale from 0 (high risk of bias) to 9 (low risk of bias).

The NOS assessed three specific criteria: selection, comparability and exposure. Only the item comparability could be awarded two points for a maximum of two adjusted confounders in the analysis. According to Araújo (25), studies presenting a summarizing score above the median are considered to have a low risk of bias. Therefore, a high risk of bias was considered in the case of a summarizing star score of < 6, and at low risk of bias was considered in the case of a star score of > 6. Quality was based on the number of stars reached. Inter-examiner agreement was ascertained through a kappa-test; any disagreement was resolved by discussion, consulting a third advisor (D.S.P.).

## Results

-Study selection 

**Figure 1:**
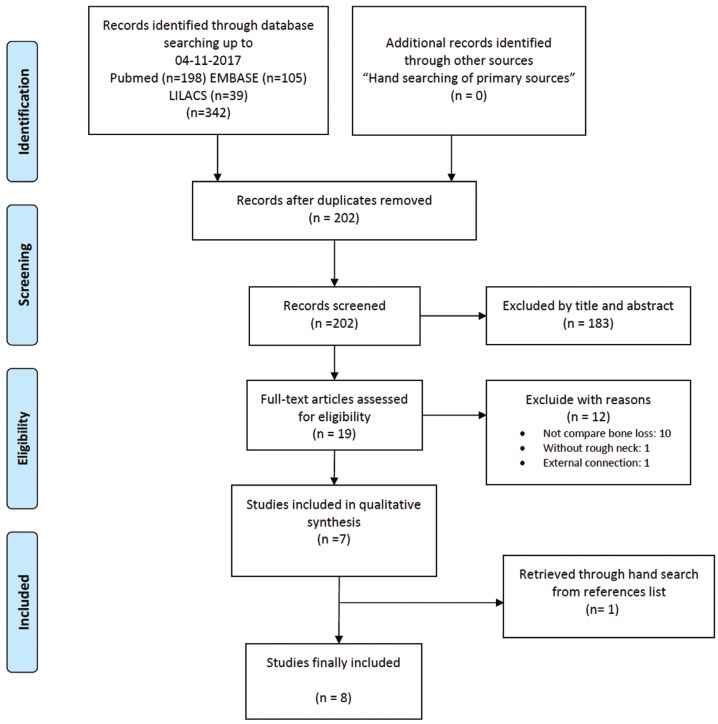
PRISMA flowchart of searching and selection process of titles during systematic review.

A total of 342 articles were obtained from the electronic search. After screening by title and abstract (interviewer agreement = 95.71%; kappa = 0.61; 95% CI [0.53–0.69]; *p* < 0.001), rejecting 183 and selecting 19 titles for full-text assessment of eligibility. Seven articles finally fulfilled the eligibility criteria; in addition, one further title was retrieved from the reference lists of included studies (interviewer agreement = 98.10%; kappa = 0.95; 95% CI [0.86–1.00]; *p* < 0.001). The reviewers agreement was substantial and almost perfect based on Landis and Koch scale. The screening process is shown in Figure 1. Of these 8 articles, 5 were RCTs (12,26-29) and three were prospective cohort studies (30-32); of the latter, two articles comprised the same prospective cohort (30,31). A summary of study characteristics is provided in  Table 1,  Table 1 continue. The excluded titles, with the reasons for exclusion, are described in  Table 2.

**Table 1:**
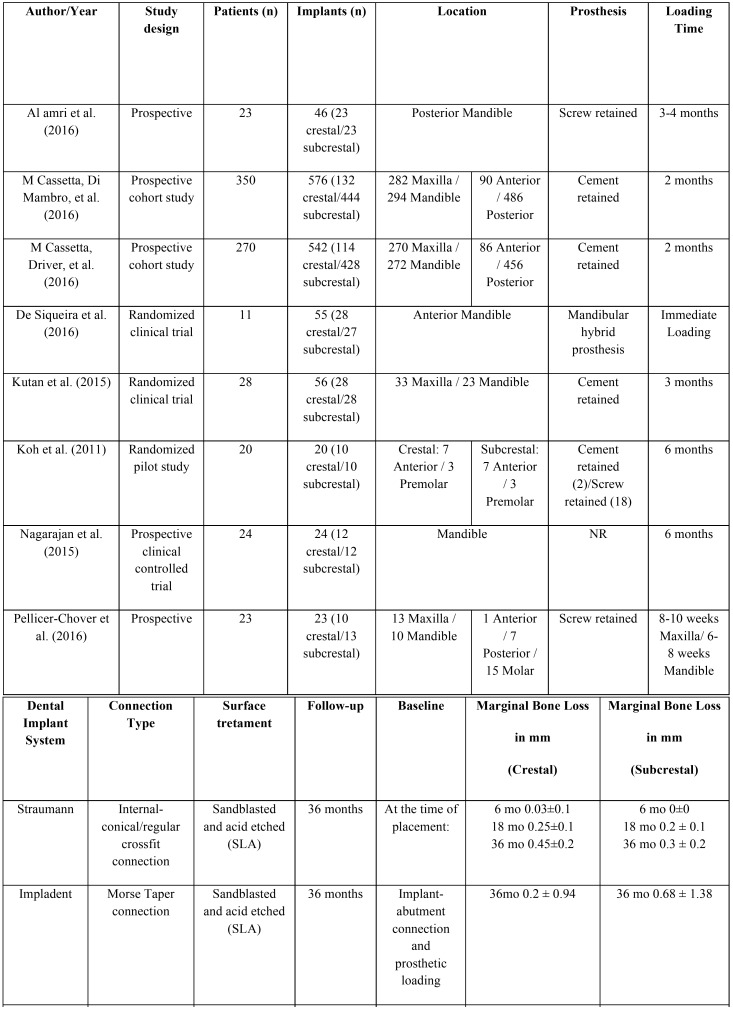
Characteristics of included studies.

**Table 1 continue:**
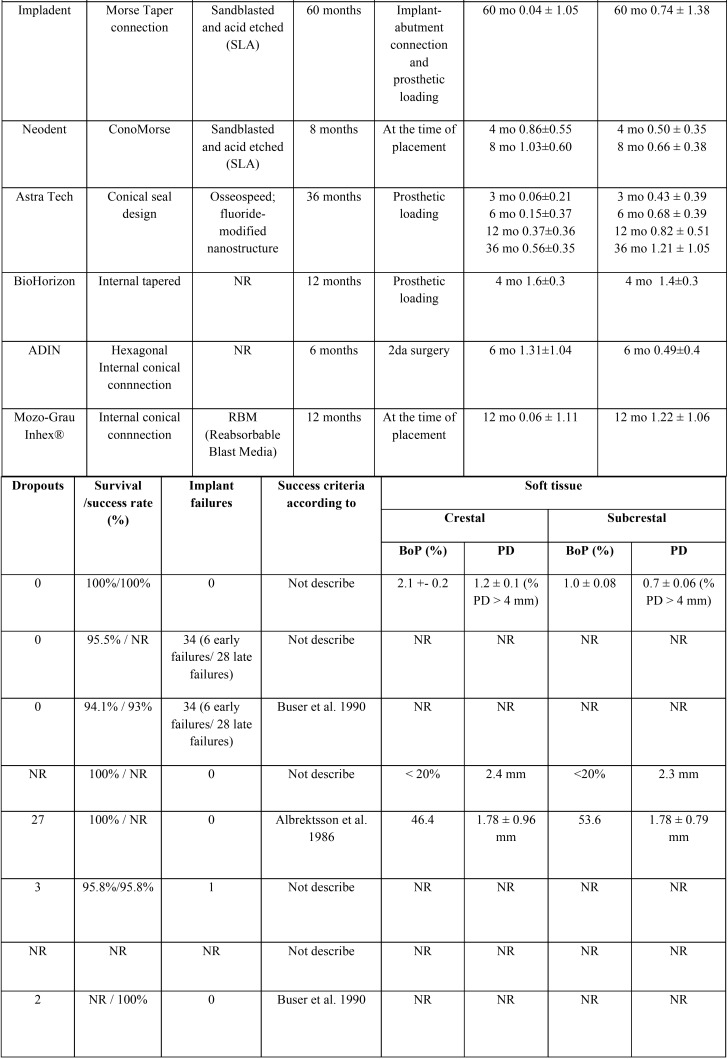
Characteristics of included studies.

**Table 2:**
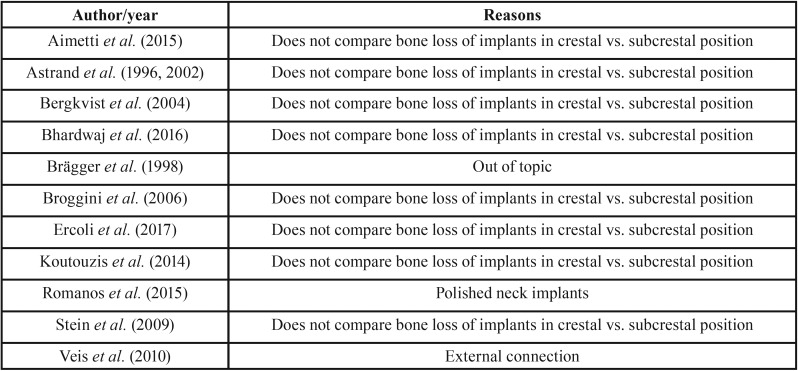
Articles excluded with reasons in the present systematic review.

-Risk of bias assessment 

**Figure 2:**
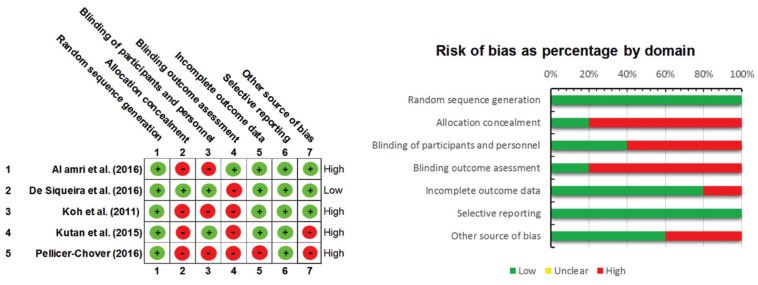
Summary of the risk of bias on the trial studies included in the systematic review according to the Cochrane Collaboration’s Tool. Low risk of bias (green); high risk of bias (red).

Inter-examiner agreement in methodological assessment was almost perfect (kappa index, k = 0.87), according to the Landis and Koch scale. The risk of bias across included titles was assessed according to the Cochrane Collaboration tool (n=5) and NOS for non-randomized studies (n=3). Several methodological flaws were identified: scantiness of data regarding allocation concealment, the blinding of participants and personnel, and the blinding of outcomes assessment, across RCTs. Only one study was considered to present a low risk of bias (27) (Fig. 2).

Regarding the observational studies, Cassetta, Di Mambro *et al.* (30) presented a score of 6 out of 9, another article (31), 5 out of 9, and one study (32), 4 out of 9. The observational studies therefore showed a high risk of bias ( Table 3).

**Table 3:**
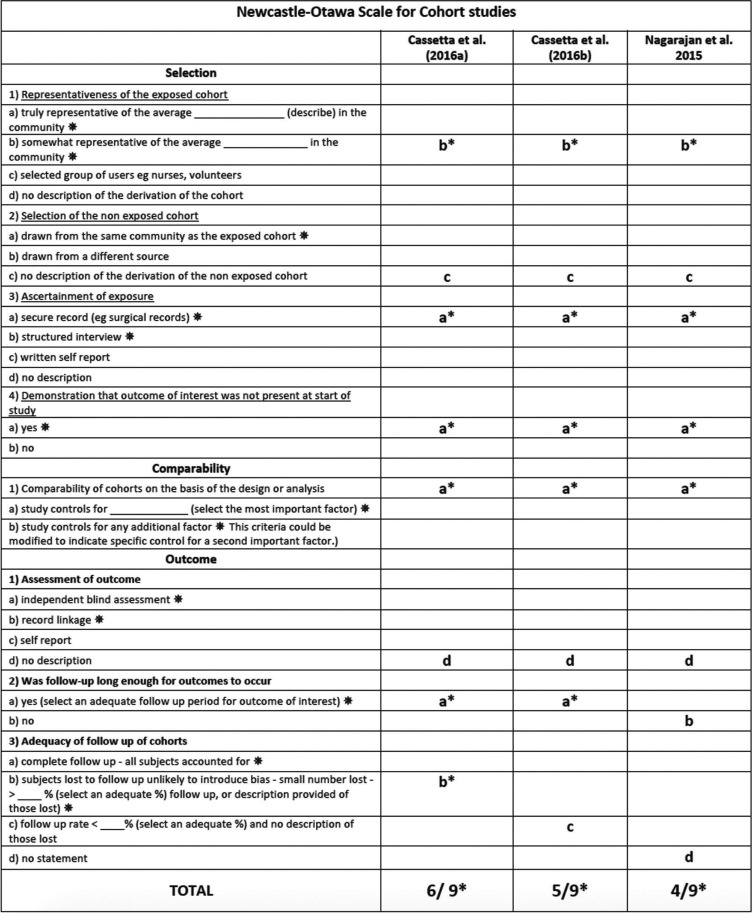
Summary of the risk of bias of the cohort studies included in the systematic review according to the NOS.

-Data extraction

The 7 selected studies (eight articles) comprised 479 patients, of which 32 could not be analyzed due to dropouts occurring during the follow-up period. A total of 800 implants were placed, of which 243 were crestal implants (30.38%) and 557 subcrestal implants (69.63%). The mean follow-up period was 21 months (range 6-36 months). Five studies (12,26,27,29,32) excluded smokers, and three articles (28,30,31) excluded patients who consumed more than 10 cigarettes a day. Six articles (12,29-32) adopted a two-stage approach, and all implants were covered with mucosa at the moment of implant placement. In contrast, one study (26) connected healing abutments to the implants at implant placement, while another study (27) used an immediate loading protocol.

The measurements of marginal bone level versus the implant shoulder varied in terms of the methodology used. In four articles (26,29-31) the mean mesial and distal marginal bone loss was expressed with positive values if the marginal bone was in a coronal position at the implant shoulder; as zero value if the marginal bone corresponded to the shoulder of the implant; and as negative values if the marginal bone was apical to the implant shoulder. The rest of the studies did not specify the methodology used for the measurement of MBL. The baseline reference of the measurements corresponded to the moment of implant placement (26,27,29-32) and the moment the prosthetic loading (12,28). Peri-implant MBL was registered in both groups in all studies. In crestal implants, MBL ranged between -0.03 and -1.6 mm, while in subcrestal implants it ranged between 0 and -1.4 mm. In four studies (26-28,32), implants placed in a crestal position presented higher MBL than subcrestal implants - the differences being significant in one study (32). On the other hand, in three studies (12,29-31), implants placed in a subcrestal position presented greater MBL than crestal implants, with significant differences in only one study (29).

All implants were restored with fixed prostheses, including screw-retained (26,28,29), cement-retained (12,28,30,31) and hybrid prostheses (27). One article (32) failed to report prosthetic restoration.

The present systematic review included studies with internal connection without platform-switching (28) and internal connection with platform-switching, comprising: conical internal connection (12,26,29), morse taper connection (27,30,31), and hexagonal internal connection (32).

Four reports offered percentage success criteria: 100% (26,29), 93% (31) and 95.8% (28). On the other hand, 6 articles reported percentage survival criteria: 100% (12,26,27), 95.5% (28), 94.1% (30) and 95.8% (30).

Three studies (12,26,27) included the analysis of periimplant soft tissues in their protocols. The variables analyzed were: probing depth (12,26,27), modified plaque index (12), keratinized tissue width and thickness (27), bleeding on probing (12,26,27) and the Löe and Silness gingival index (12). No differences in the variables analyzed were observed between the crestal and subcrestal groups.

## Discussion

The present systematic review aimed to compare peri-implant bone changes in internal connection implants with a rough neck and with or without platform-switching placed in a crestal versus subcrestal position in clinical studies. The review comprised data from 8 articles corresponding to 7 studies: 5 experimental and two observational (cohort studies). The prospective cohort study (30,31) reported results at different time intervals of 36 and 60 months, respectively. Thus, we considered assessment and summary of each article as an individual study. Only one RCT showed a low risk of bias (27). Observational studies presented high risk of bias according to the NOS (23). Allocation concealment, the blinding of participants and outcomes assessor were limitations detected among studies, as well as inadequate description of the non-exposed cohort in observational studies, and attrition bias with a dropout rate of > 20% in one study (31).

The placement of an implant in a subcrestal position has been suggested as a method that could contribute to the maintenance of hard and soft periimplant tissues compared to a crestal placement – though this affirmation is subject to debate. Experimental animal studies (14,17,33) and human studies (13,15,34) involving polished neck implants have observed that subcrestal implant placement produces an increase in peri-implant bone loss. Various experimental studies in animals (35,36) have found that low surface roughness (Sa value 0.5-1 μm), such as that found in polished neck implants, promotes the formation of fibrous capsules around the polished surface of the implant and produces a smaller bone-implant contact area. Conversely, some authors (37-39) found that osseointegration could occur on the implant platform when positioned 2 mm subcrestal in implants with a rough neck design and platform-switching. The latter is a design where the diameter of the abutment is smaller than the neck of the implant. Such designs have been associated with a decrease in periimplant bone loss compared to standard platform implants, thanks to the internally repositioned implant-abutment junction (mismatching), which limits periimplant bone loss by distancing bacteria and infiltrating inflammatory cells away from adjacent crestal bone (6). The differences in bone loss results among authors can also be attributed to the type of prosthetic connection or the type of restoration involved (19). A recent systematic review (40) claimed the superiority of conical connections in sealing, microgap formation, torque maintenance and stability of the prosthetic abutment. These finding suggest that macro- and micro-designing of the implant could play an important role in marginal periimplant bone changes when the implants are placed subcrestally.

The results of our systematic review showed exposed rough surface around subcrestal implants to less pronounced than in the case of crestal implants. In crestal implants, periimplant bone remodeling immediately results in exposure of the rough surface of the implant. This does not happen when the implants are placed subcrestally, since the starting point of bone is above the implant platform, and the surface is contained within the periimplant defect produced by drilling. Therefore, bone remodeling does not necessarily lead to exposure of the rough surface of the implant. This fact justifies the study of the variable “exposed rough surface”. Kütan *et al.* (12) found mean radiographic vertical bone loss in the crestal group after three years to be significantly smaller than in subcrestal group (0.56±0.35 mm and 1.21±1.05 mm, respectively), though reabsorption did not reach the implant threading. In the control group, the first bone-implant contact was located under the level of the first threads. Pellicer-Chover *et al.* (29), after 12 months of follow-up, recorded a bone loss of 0.06 mm in crestal implants versus 1.22 in subcrestal implants. However, on analyzing the exposed rough surface, the subcrestal implants presented lower values (mean 0.57 mm) than the crestal implants (mean 1.13 mm). In this same line, Al Amri *et al.* (30) observed that in contrast to implants placed subcrestally, crestal implants presented bone levels below the platform (-0.45±0.2 mm), and therefore exposure of the rough surface of the implant. Since exposed surfaces of the implants could lead to complications in peri-implant health, the authors suggested that subcrestal placement of the implants is preferable.

Four studies reported success criteria of between 93-100%. In turn, the success/survival criteria across the included studies are consistent with those reported by Albrektsson *et al.* (1) and Buser *et al.* (35) ( Table 1. Among the 6 articles that reported implant survival, the range was 94.1-100%. Six early failures before loading and 28 late failures after loading were described - in both cases secondary to peri-implant tissue infection (30,31).

The results referred to soft tissue outcomes in the present study should be interpreted with caution, since such soft tissue measurements were reported in only three studies (12,26,27), and with important heterogeneity in the approaches used to assess the parameters among studies. Al Amri *et al.* (26), in crestal implants, found the highest mean percentage of sites with bleeding on probing and probing depth ≥ 4 mm to be recorded at 6 months (7.4% and 1.4%, respectively). In subcrestal implants, the highest mean percentage of sites that showed bleeding on probing and probing depth ≥ 4 mm was recorded at 6 months (2.4% and 1.2%, respectively). In contrast, Kütan *et al.* (12) and De Siqueira *et al.* (27) reported no statistically significant differences between the two groups in terms of periodontal indexes.

Study strengths, limitations and recommendations

To the best of our knowledge, this is the first review that assesses the impact of the positioning of crest and subcrestal implants in the neck and platform on marginal bone loss. Due to the variability in the design and execution of the studies, the present work tries to provide information for improvement in future studies.

The limitations of our study include the difficulty of obtaining data on the type, design (polished or treated neck) and connection (conical design, with or without platform-switching) of the implants used in each study – this resulting in a lack of information that is reflected in the results of the systematic review. The articles included showed variability in their way of measuring marginal bone loss. In this regard, when implants are placed subcrestally it may be advisable to report the measurements as positive values when the bone is above the platform and as negative values when below the platform.

Likewise, significant differences were observed in the moment of starting to measure bone loss (baseline) - a fact that could result in erroneous data. Seven articles (26,27,29-32) defined the moment of implant placement as the starting point, while two studies (12,28) started measurement at the time of prosthetic loading.

Unfortunately, human studies evaluating the effects of apico-coronal implant placement on postsurgical marginal bone loss are limited. This may be due in part to the strict inclusion criteria applied in an attempt to provide direct and less biased comparisons. Further studies on this subject are required, in view of the lack of standardization found in the articles included in our review.

Future studies in this field are needed to overcome the methodological shortcomings, specifically in relation to allocation concealment and blinding of the participants in RCTs, with better sample size estimations and adequate statistical power, in order to confirm the trends observed in our review. Such studies moreover should also address the impact of other risk factors or modifiers such as smoking, alcohol, or controlled systemic diseases such as type 2 diabetes.

Despite its limitations, the present systematic review did not find better outcomes between crestal and subcrestal implant placement. In four studies, implants placed in a crestal position presented higher MBL than subcrestal implants - the differences being significant in one study, while in three studies, implants placed in a subcrestal position presented greater MBL than crestal implants, with significant differences in only one study. The underlying evidence is limited and of low quality, so to confirm this finding and determine whether it is clinically relevant, new studies are needed, involving improved designs and the standardization of protocols of variable assessment to allow statistical comparisons. Further clinical studies with longer follow-up times and larger sample sizes are required to improve our understanding of this interesting and frequent topic in clinical practice.
